# Whole-genome characterization and phylogenetic analysis of Equine Rotavirus A isolated from Jeju Island, Korea

**DOI:** 10.4142/jvs.25333

**Published:** 2026-05-26

**Authors:** Thanh Tam Pham, Dong-Ha Lee, Eun-Ju Ko

**Affiliations:** College of Veterinary Medicine and Veterinary Medical Research Institute, Jeju National University, Jeju 63243, Korea.

**Keywords:** Horses, rotavirus A, molecular epidemiology, reassortment, genetic, genome, viral

## Abstract

**Importance:**

Equine Rotavirus A (ERVA) is a major cause of acute diarrhea in neonatal foals, leading to significant economic losses. Genetic variation and global transmission complicate disease control efforts.

**Objective:**

To analyze the complete genome of an ERVA strain from Jeju Island, Korea, and assess its genetic and phylogenetic relationships with global ERVA strains.

**Methods:**

Twenty-five fecal and swab samples were collected from diarrheic horses in Jeju during spring and summer 2025. Samples were screened using antigen testing and RT-PCR. One PCR-positive, antigen-test-positive sample was selected for whole-genome sequencing. All 11 segments were amplified using segment-specific primers and sequenced by the Sanger method.

**Results:**

The Jeju isolate exhibited a G14-P[12]-I2-R2-C2-M3-A10-N2-T3-E2-H7 genome constellation, matching the globally prevalent ERVA genotypes. Most segments showed > 99% identity to a South African strain (SA1), while the VP4 segment showed higher similarity to a Japanese BI strain, suggesting a reassortment event. Phylogenetic analysis grouped the Jeju strain with isolates from South Africa and Ireland, but distinct from recent Japanese strains, indicating independent transmission patterns.

**Conclusions and Relevance:**

This is the first full-genome report of ERVA from Korea. The findings confirm the presence of globally related ERVA in Korean horses and suggest complex, international transmission dynamics. Continuous molecular surveillance is essential to support effective prevention and vaccine strategies.

## INTRODUCTION

Equine Rotavirus A (ERVA) is the leading cause of acute dehydrating diarrhea in newborn foals, primarily affecting those under six months of age [1]. The disease progresses rapidly, leading to severe dehydration and electrolyte imbalances, which can be fatal without timely intervention [1]. ERVA primarily damages the small intestine, impairing absorption and digestion; however, recovery is possible through epithelial cell turnover [1,2].

ERVA is globally distributed, with cases reported across multiple continents [3,4]. Transmission occurs through the fecal-oral route. Viral shedding can last up to seven days post-recovery, and virions can remain viable in the environment for several months [1]. Notably, asymptomatic mares can also acquire ERVA from infected foals, contributing to further environmental spread [1].

ERVA belongs to the genus Rotavirus A (RVA), characterized by a double-stranded RNA genome with 11 segments and a triple-layered capsid. VP7 and VP4 are outer layer capsid proteins that induce the major neutralizing antibodies and are thus used to classify ERVA into G-types and P-types, respectively. Genotypes G3P[12] and G14P[12] are the most detected and epidemiologically relevant worldwide [5,6,7,8,9,10]. The prevalence of G3 and G14 strains fluctuates over time due to changing herd immunity and global horse trade [2,5,6,7,8,10,11]. The predominant ERVA strains exhibit a unique genotype constellation (G3/G14-P[12]-I2/I6-R2-C2-M3-A10-N2-T3-E2/E12-H7), that is distinct from non-ERVA strains [9,12]. Unusual genotype constellations showing similarity to rotaviruses from other species, such as porcine, bovine, and feline, have also been detected, implying cross-species infection [1,4,11,13].

Vaccination is the primary preventive measure, typically administered to pregnant mares to enhance passive immunity in foals [1]. Although inactivated vaccines based on the G3P[12] strain have been developed in the USA and Japan and Argentina [13], none are currently licensed or commercially available in South Korea. Despite high concentrations of neutralizing antibodies in maternal sera, vaccine breakthroughs due to G14P[12] strains have been reported [4]. Consequently, multivalent vaccines incorporating both G3 and G14 may provide broader protection.

While Japan has conducted extensive molecular studies on ERVA, many regions in Asia still lack sufficient data [4,9,10,12]. Jeju Island, the major horse farming hub in Korea, has reported ERVA cases; however, further molecular research is essential. Therefore, this study aims to analyze the complete genome of an ERVA strain from Jeju Island to elucidate its molecular epidemiology and enhance disease management and vaccine strategies.

## METHODS

### Sample collection

Twenty-five fecal and swab samples were collected from nine diarrheic foals and sixteen diarrheic adult horses on Jeju Island, South Korea, from spring to summer of 2024. Each sample was diluted in phosphate-buffered saline, filtered and stored at −80°C until further processing.

### Antigen and reverse transcription-polymerase chain reaction (RT-PCR) screening

All the samples were tested using both an antigen test and RT-PCR. The antigen test was conducted with the Asan Easy Test ROTA Strip (Asanpharm, Korea) according to the manufacturer’s instructions. Viral RNA was extracted using the QIAamp Viral RNA Mini Kit (Qiagen, Germany) following the manufacturer’s protocol, and then subjected to RT-PCR using the Beg9-End9 primers as previously described [7]. Samples confirmed as positive by RT-PCR were selected for full-genome sequencing.

#### Sequencing and genome assembly

All 11 segments of the rotavirus genome were amplified by RT-PCR using segment-specific primers as described previously [9], with minor modifications as detailed in Supplementary Table 1. RT-PCR were carried out using the Maxima™ H minus cDNA synthesis master mix (Thermo Fisher Scientific, USA) and Q5 master mix (NEB, USA) to maintain high fidelity. The polymerase chain reaction (PCR) products were analyzed via agarose gel electrophoresis and then purified using the QIAquick PCR Purification Kit (Qiagen). Subsequently, 150 ng of the purified PCR products were submitted to a commercial sequencing service (Macrogen, Korea) for sequencing according to the provider’s standard Sanger sequencing protocol. The VP1, VP2, VP3 sequences were further determined by primer walking with primers designed from the preceding sequencing runs (primers are provided in Supplementary Table 1). The resulting sequences were assembled and edited using MEGA11 [14] and manually checked for accuracy.

#### Genetics analysis

Each genomic sequence was genotyped using the National Institute for Public Health and the Environment (RIVM) Rotavirus Genotyping Tool [15], which assigns genotypes based on sequence similarity to curated reference strains [16]. All 11 sequences were compared to reference strains in the National Center for Biotechnology Information (NCBI) nucleotide database using BLASTn [17]. Percent identity scores were used to assess the relatedness between the Jeju isolate and other known ERVA strains.

#### Phylogenetic analysis

Phylogenetic trees were constructed for each sequence using MEGA11 software [14]. Sequences were aligned with ClustalW, and evolutionary trees were generated using the Maximum Likelihood method with 1,000 bootstrap replicates. Evolutionary distances were calculated using the Maximum Composite Likelihood method, and ambiguous positions were removed using the pairwise deletion option. A total of 26 ERVA reference sequences were included in the analysis, sourced from NCBI.

### Mutation analysis

The structure of VP4 was predicted using ColabFold [18] and the models were superimposed using ChimeraX. Molecular graphics and analyses were performed with UCSF ChimeraX, developed by the Resource for Biocomputing, Visualization, and Informatics at the University of California, San Francisco, with support from National Institutes of Health R01-GM129325 and the Office of Cyber Infrastructure and Computational Biology, National Institute of Allergy and Infectious Diseases.

## RESULTS

### Detection of RVA in clinical samples

**Fig. 1:**
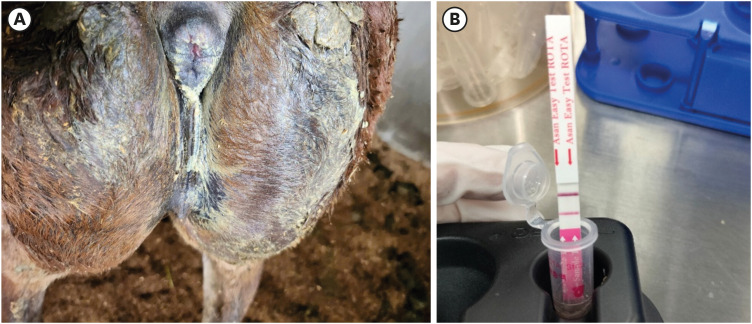
Clinical and diagnostic identification of ERVA in a foal. (A) Clinical presentation of ERVA infection: Explosive diarrhea symptoms observed in a newborn foal selected for sample collection on Jeju Island. Severe watery diarrhea is a hallmark of ERVA, which can lead to rapid dehydration and electrolyte imbalances. (B) Rapid antigen detection: Result of the Asan Easy Test ROTA strip used for pre-screening. The lower band represents internal control, and the upper band indicates a positive result for the rotavirus antigen in the fecal sample. While utilized as a primary screening tool, the positive result was further confirmed by reverse transcription-polymerase chain reaction for definitive genomic characterization. ERVA, Equine Rotavirus A.

Among twenty-five samples were collected, two samples were RVA positive by RT-PCR and one of these samples was RVA-positive by the antigen test. This sample was obtained from a diarrheic foal, exhibiting explosive diarrhea (Fig. 1).

### Genotype constellation of the Jeju isolate

All 11 segments were sequenced and the nucleotide sequences were submitted to GenBank (PV863435-PV863445). Analysis using the RIVM genotyping tool [16] identified the isolate as having the genotype constellation G14-P[12]-I2-R2-C2-M3-A10-N2-T3-E2-H7.

### Molecular and phylogenetic characterization of the Jeju isolate

**Fig. 2:**
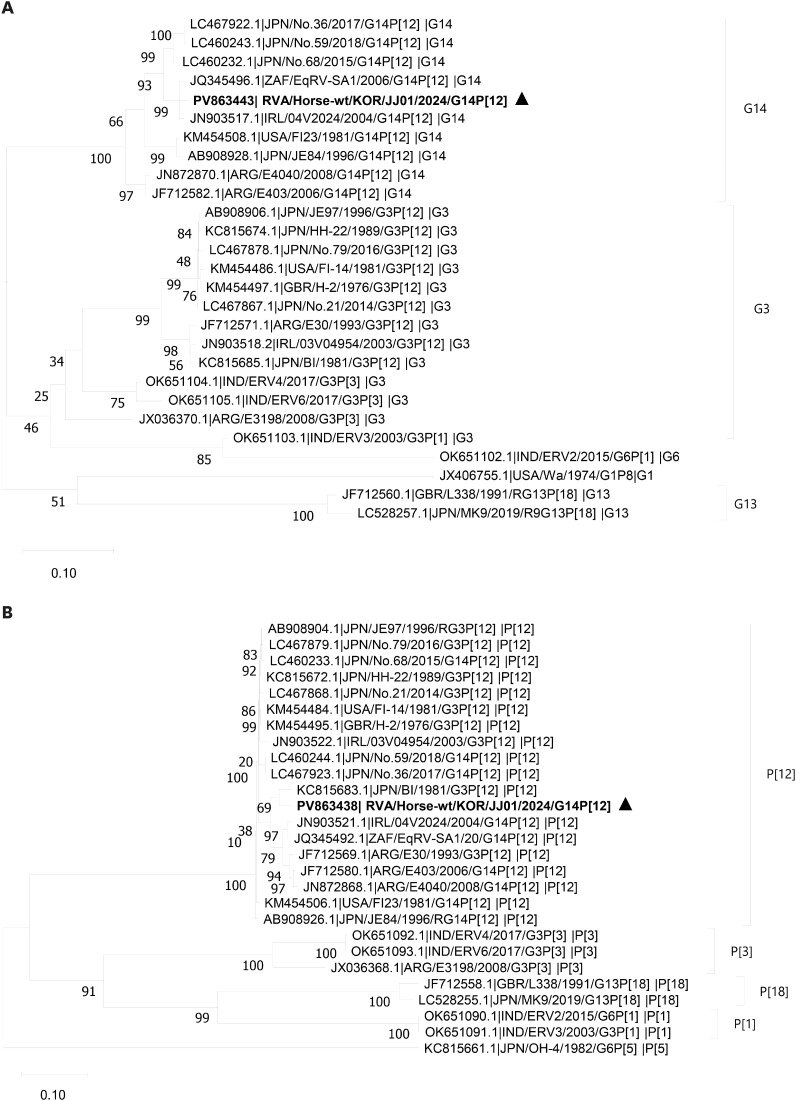
Phylogenetic analysis of the VP7 and VP4 segments of the Jeju ERVA isolate (JJ01). (A) Phylogenetic tree of the VP7 sequence: The evolutionary history was inferred using the Maximum Likelihood method and the Tamura-Nei model. The Jeju isolate (JJ01, indicated in bold and ▲) clusters within the G14 lineage, showing close proximity to South African and Irish strains. (B) Phylogenetic tree of the VP4 sequence: The evolutionary history was inferred using the Maximum Likelihood method and the Hasegawa-Kishino-Yano model. The JJ01 strain (indicated in bold and ▲) is located within the P[12] genotype cluster. For both trees, the percentage of trees in which the associated taxa clustered together is shown below the branches (1,000 bootstrap replicates). The trees are drawn to scale, with branch lengths measured in the number of substitutions per site. All evolutionary analyses were conducted in MEGA11.

Each sequence of the Jeju isolate was aligned using the BLAST tool against the NCBI database. All the nucleotide sequences showed > 99% identity with the corresponding sequences of the South African isolate RVA/Horse-wt/ZAF/EqRV-SA1/2006/G14P[12], with the exception of the VP4 sequence (Supplementary Table 2). The whole genome phylogenetic analysis based on 26 available ERVA sequences from the NCBI database, placed the Jeju isolate in the same clade as the South African strain (2006) and the Irish isolate (2004). The Jeju strain was relatively distinct from other ERVA isolates, particularly recent Japanese strains (Fig. 2, Supplementary Figs. 1, 2, 3).

Segment-specific phylogenetic analyses were performed for the Jeju isolate. Shorter segments lacking sufficient phylogenetically informative mutations were excluded from further analysis. The phylogenetic trees for segments VP1, VP2 and VP3 (Supplementary Figs. 1, 2, 3) showed that the Jeju isolate clustered with the isolates from South Africa (2006) and Ireland (2004).

VP4 and VP7 which are the principal neutralization antigens of RVA. Therefore, mutations in these sequences can drive genetic drift and have clinically and epidemiologically significant. Phylogenetic analysis of the VP7 sequence showed a similar clustering pattern to the other segments and the whole genome (Fig.2A). In contrast, the VP4 sequence analysis revealed a different pattern. Although most of VP4 sequences belong to P[12] genotype, the clades contained both G3 and G14 strains and tended to cluster by sample location and year rather than the G-type (Fig. 2B). The segment 4 of the Jeju isolate showed 97% identity to the Japanese BI isolate (RVA/Horse-tc/JPN/BI/1981/G3P[12]) and 96% identity to the SA1 isolate (Supplementary Table 2). The Jeju isolate clustered within the same clade as the Japanese BI isolate, differing from the clustering observed in the other 10 segments.

**Fig. 3:**
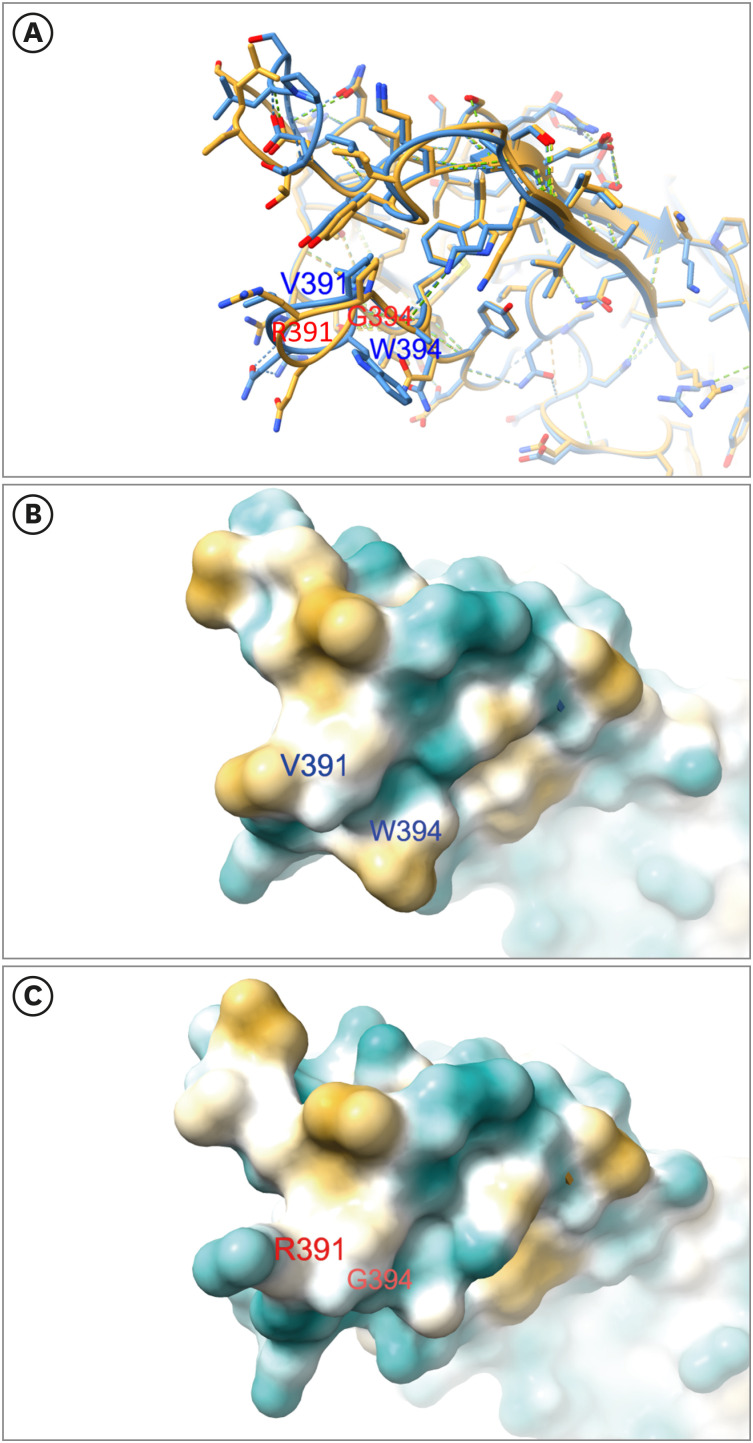
Structural modeling and surface analysis of the VP5 protein in the JJ01 isolate. (A) Comparative structural model: Superimposition of the VP5 loops from the SA11 strain (blue) and the Jeju isolate JJ01 (yellow). The two non-conservative mutations, V391R and W394G, are highlighted. (B) Hydrophobic-hydrophilic surface model of SA11: In the reference strain, the loop features two distinct hydrophobic protrusions (V391 and W394). (C) Hydrophobic-hydrophilic surface model of JJ01: The mutations replace the hydrophobic protrusions with hydrophilic side chains (indicated in red labels). These biochemical changes at the top of the loop are hypothesized to alter the membrane penetration capability of the VP5 spike, potentially affecting viral infectivity.

Further analysis of the VP4 protein sequence of the Jeju isolate identified nine unique amino acid substitutions. Seven of these were conservative, while two mutations were non-conservative: V391R and W394G. The structure of VP5 from the Jeju isolate, predicted by ColabFold, was compared with the structure of SA11 strain (Uniprot Identifier 2B4I) (Fig. 3). The comparison revealed that V391R and W394G replaced two hydrophobic protrusions of a loop with two hydrophilic side chains.

## DISCUSSION

The ERVA isolate from Jeju Island exhibits a genotype constellation of G14-P[12]-I2-R2-C2-M3-A10-N2-T3-E2-H7, which is consistent with the most commonly reported ERVA genotypes globally. This study confirms the presence of RVA and represents the first full-genome sequence analysis of an ERVA strain collected from horses in Korea.

Phylogenetic analysis demonstrated a close relationship between the Jeju isolate and strains from South Africa (2006) and Ireland (2004), but revealed limited similarity to recent Japanese isolates. This suggests minimal RVA exchange between Japan and Jeju, despite their geographical proximity, and points to complex global transmission dynamics.

Interestingly, the VP4 sequence displayed greater phylogenetic diversity than other segments, often clustering by year and location rather than by G type. This observation suggests frequent reassortment events between G3 and G14 strains. Overall, the Jeju ERVA isolate appears to be a reassortant virus that combines a conserved backbone with a divergent VP4 segment, emphasizing the importance of whole-genome monitoring to detect emerging variants that may influence pathogenicity and cross-regional spread.

Furthermore, within VP4 sequence, two amino acid substitutions (V391R and W394G) were mapped to the hydrophobic loop of the VP5 spike, a region known to mediate membrane penetration. Structural modeling predicted that these changes replace hydrophobic residues with hydrophilic side chains, which may alter lipid bilayer interaction We hypothesize that these substitutions could potentially reduce viral infectivity, as similar biochemical alterations in the hydrophobic loop have been reported to impair membrane permeabilization in other RVA strains [19]. Therefore, continuous surveillance of VP4 variability and subsequent in-depth functional assays are essential for a better understanding of ERVA evolution and the development of effective control strategies.

## SUPPLEMENTARY MATERIALS

Supplementary Table 1 Primers used for reverse transcription-polymerase chain reaction amplification and sequencing of the 11 genomic segments of Equine Rotavirus A (JJ01)

Supplementary Table 2 Nucleotide sequence identities (%) of the 11 genomic segments between the Jeju isolate (JJ01) and representative Equine Rotavirus A strains

Supplementary Fig. 1 Phylogenetic analysis of the segment encoding VP1 of Equine Rotavirus A. The evolutionary history was inferred using the Maximum Likelihood method and the General Time Reversible model. The Jeju isolate (JJ01, indicated in bold and ▲) clusters within the R2 genotype, showing a close genetic relationship with strains from South Africa (2006) and Ireland (2004). The percentage of trees in which the associated taxa clustered together is shown below the branches (1,000 bootstrap replicates). The tree is drawn to scale, with branch lengths measured in the number of substitutions per site. Evolutionary analyses were conducted in MEGA11.

Supplementary Fig. 2 Phylogenetic analysis of the segment encoding VP2 of Equine Rotavirus A. The evolutionary history was inferred using the Maximum Likelihood method and the General Time Reversible model. The Jeju isolate (JJ01, indicated in bold and ▲) clusters within the C2 genotype, showing high similarity to strains from South Africa and Ireland. The percentage of trees in which the associated taxa clustered together is shown below the branches (1,000 bootstrap replicates). The tree is drawn to scale, with branch lengths measured in the number of substitutions per site. Evolutionary analyses were conducted in MEGA11.

Supplementary Fig. 3 Phylogenetic analysis of the segment encoding VP3 of Equine Rotavirus A. The evolutionary history was inferred using the Maximum Likelihood method and the General Time Reversible model. The Jeju isolate (JJ01, indicated in bold and ▲) clusters within the M3 genotype. The percentage of trees in which the associated taxa clustered together is shown below the branches (1,000 bootstrap replicates). The tree is drawn to scale, with branch lengths measured in the number of substitutions per site. Evolutionary analyses were conducted in MEGA11.
